# Information Typology in Coronavirus (COVID-19) Crisis; a Commentary 

**Published:** 2020-03-12

**Authors:** Hasan Ashrafi-rizi, Zahra Kazempour

**Affiliations:** 1 Medical Library and Information Science Department, Health Information Technology Research Center, Isfahan University of Medical Sciences, Isfahan, Iran.; 2 Library and Information Science Department, Faculty of Media, Payame Noor University, Tehran, Iran.

**Keywords:** Coronavirus, Disaster Planning, Confidentiality, Information Typology, Access to Information

## Introduction:

In late 2019 and early 2020, many people in different countries around the world became infected by the new Coronavirus. This created challenges for these countries in many aspects including economic, political, social, health and so on. Some of these challenges are directly or indirectly related to information discussion, because providing the right information, at the right time and to the right audience, can solve or reduce some of the challenges. However, there were problems in this process during this crisis, as various individuals and organizations began to produce and disseminate information that, given the special circumstances of this crisis (that most countries have rarely experienced), produced types of information that are worth consideration. 

## Methods:

In a commentary, the author(s) seek to present new views to researchers on a particular topic. A commentary may also draw attention to current advances and speculate on future directions of a certain topic (1). Accordingly, the authors of this commentary began to review scientific texts and messages published in the mass and social media on the coronavirus. Also, authors used previous knowledge and experience, particularly in the areas of health information behavior, health information literacy, and media literacy. The messages were analyzed and categorized by the authors, and the information typology was selected based on the categories of the messages and their context. In addition, the views of two other researchers on content and terms were also obtained. In this commentary, the authors attempted to take into account any information produced and published during the coronavirus crisis.

**Types of information:**

***Valid Information***

These are information that is based on the latest scientific evidence and is citable and applicable to others. For example, hand washing with a certain protocol can lead to elimination of the coronavirus. 

***Comforting Information***

 These are information whose production and dissemination makes people happy and relaxed during the crisis. In the current coronavirus crisis, jokes, animations, poem manipulation, mass media entertainment and other content that decreased an individual's fears and anxieties about the coronavirus for moments, were instances of comforting information that act like a safety valve.

***Perplexing information***

 The kind of scientific information that has been produced in order to increase the knowledge of others, but is sent to an unrelated audience, is called perplexing information. For example, some high-level scientific information about coronavirus is sent to the general public or adolescents, who do not have enough knowledge and cannot understand it, which can in turn exacerbate their concerns. On the other hand, sometimes some simple content is sent to health professionals, for whom the content is elementary, and this can lead to wasting their time. Most of this content is shared on online social networks.

***Misinformation***

This type of information is inaccurate and unreliable but the distributer of that information disseminates it inadvertently and pays little attention to the effects of this information on the society (2). In the Coronavirus crisis, bad news that was spread through online social networks such as WhatsApp, Telegram, etc. regarding the number of infected and dead people increased the fear and anxiety among people. However, if this kind of news is produced and distributed on purpose, it is no longer called misinformation; it is named disinformation because the producer(s) pursued inhumane purposes.

***Disinformation***

This is the type of inaccurate information whose producers and distributors pursue political, economic, cultural, or other purposes and intentionally produce and disseminate it. This type of information is intentional, forging, and manipulative and distorts reality (2). This kind of information is usually produced and disseminated by rancorous people. For example, a message on the social media stated that a Bahraini officer said that the Iranian president and all his cabinet members were suffering from coronavirus disease. 

***Shocking Information***

Reading or hearing this type of information makes the recipient consternated, shocked and anxious. For example, in the early days of the Coronavirus crisis, information that illustrated the dangerous nature and behavior of the virus, like its contagiousness, was sent to the world from china. This information was unbelievable and terrifying to the general public.

***Contradictory Information***

 This type of information is produced and disseminated due to a difference of opinion between experts on a topic. In the current Coronavirus crisis, some experts initially declared that using a mask in the public was necessary, but some disagreed. Some even stated that the World Health Organization has behaved differently in this case.

***Doubtful (Untrusted) Information***

This type of information cannot be validated or discredited due to insufficient scientific evidence. For example, in case of the new coronavirus, some have claimed that peganum smoke and consumption of garlic or other foods are helpful in disease prevention, and some have dismissed their effectiveness. The accuracy of these claims should be scientifically evaluated by health researchers.

***Progressive Information***

This type of information fosters or leads to innovation, creativity, and production of new content in the future. For example, information obtained by health researchers about the nature and function of the virus during the coronavirus crisis will be the basis for future studies. This is the reason that it has been stated that the choice of research topics should be based on need. In fact, it is an example of research based on society's needs.

***Postponed Information***

 This information is presented to others with delay. If information is temporarily withheld, it will result in a consequence like distrust in society. For example, some countries initially did not disclose the number of cases infected with coronavirus, but with the increasing number of patients, they were forced to provide information.

***confidential Information***

 This type of information is never revealed for various reasons and there is deliberation and expedience in hiding them. It is clear that many countries will not disclose the exact number of infected and death cases, because a greater number indicates the weakness of the governments and their health structure. 

## Conclusion:

This will not be the first and last pandemic crisis in the world. It is important for a community to know how to manage and steer through that crisis and experts should develop appropriate behavioral patterns for both people and professional as well as government officials by gaining experience from this crisis and similar ones over the time. The proper behavioral pattern helps the community to get through the crisis with minimum cost (material and spiritual). In this behavioral pattern, attention must be paid to producers and disseminators of information, including the media, government officials, professionals in various fields (physician, nurses, psychologists, etc.) and even the public. Media professionals and medical librarians, can play an important role in finding the best behavioral model for dealing with crises due to having the necessary knowledge and awareness about information production and dissemination infrastructure and familiarity with the typology of information. In the field of prevention, teaching information literacy and health literacy, and explaining appropriate behavior in times of crisis are other actions that media professionals and medical librarians should consider. Finally, educating people on differentiating credible information from unreliable information will be the most important action taken by librarians and informants in the face of crises. It is hoped that this crisis experience will encourage medical librarians to become more active and have a more effective presence in future disasters. In conclusion, it should be mentioned that some of these types are based on the authors' understanding and inference, so everyone is welcome to help the authors improve these concepts. At the same time, lack of relevant information resources and categorization of these contents are some of the limitations of this study. Therefore, generalization of findings should be made more carefully.
